# Formulation and Delivery Systems of Cocaine and Methamphetamine Scent Mimics

**DOI:** 10.1155/ianc/2222059

**Published:** 2026-02-04

**Authors:** Silvia T. Mo, Issac C. C. Cheng, H. T. Henry Chan, Kin Yat Tong, Zhengpei Li, Yang Liu, Kelvin S. Y. Leung, Kangning Ren, Catherine H. H. Hor

**Affiliations:** ^1^ Department of Chemistry, Hong Kong Baptist University, Hong Kong SAR, China, hkbu.edu.hk

## Abstract

Narcotic scent mimics, which are chemicals that emit a perceivable scent similar to that of the illicit drug but do not possess the health and addiction risks of the drug, have applications ranging from canine training in law enforcement agencies to antinarcotic public education. However, low‐cost and easily accessible formulation and delivery systems for narcotic scent mimics have thus far been lacking. In this work, we assess the potential of synthetic (metal‐organic framework, MOF) and natural (cotton) materials as carrier and delivery agents for pseudoscent chemicals mimicking cocaine and methamphetamine. Through liquid chromatography‐based analysis, both materials demonstrate capabilities to act as inert absorbents for the scent mimics, with cotton being the more effective material. Cotton material provides a safe, durable and inexpensive medium for pseudoscent storage and release. The pseudoscent delivery systems are easy to formulate, store and distribute, thus enabling widespread use for both specialised training and public educational purposes.

## 1. Introduction

Drug abuse is a severe social and economic problem that troubles many countries globally. In 2024, the most common illicit drugs in Hong Kong included heroin, cocaine and methamphetamine [1]. Among them, cocaine is a vehemently addictive stimulant narcotic that causes permanent damage to brain tissues [2]. Methamphetamine is also an extremely addictive psychostimulant [3], commonly synthesised from phenylacetone or from over‐the‐counter medications such as ephedrine and pseudoephedrine [4–7]. Drug addiction not only causes harm to the taker’s physical health, but also causes mental health problems, including sadness, anxiety and psychosis, along with socioeconomic impact, including productivity loss, increased debts and medical costs, and violent crime [3, 8, 9]. The global extent of illicit drug use was highlighted in the United Nations’ World Drug Report 2023 [2], which described ‘a prolonged surge in both supply and demand of cocaine’ and reported methamphetamine as the primary drug declared by people entering drug treatment in East and Southeast Asia [6].

Two common strategies to fight against illicit drug use are law enforcement and seizure during transit, and public education. For drug detection, police and customs officials often deploy service dogs that have been rigorously selected and trained to search for, detect, identify and signal about the presence of narcotics, utilising the much lower odour detection threshold of dogs than humans [10–12]. Furthermore, governments often conduct antidrug campaigns which aim to increase awareness of the harm of narcotics, through exhibitions, roadshows and workshops. Acquainting the participants with the scents of narcotics would be an engaging and effective strategy, as previous studies have exemplified the appeal of olfactory experiences to interact with the participants [13, 14] and the unpleasant nature of narcotic scents to the public [15]. Moreover, by familiarising the public with narcotic scents, one can more easily detect potential drug abuse within their home and school communities, allowing especially young drug users to be identified early and cared for. Both specialised canine training and general educational campaigns would benefit from the availability of narcotic scents. Nevertheless, given the tight control of narcotic substances, the use of authentic samples in these settings bears significant security and health risks, especially in public events.

Narcotic scent mimics, or pseudoscents, offer an appealing alternative. They are substances or mixtures of chemicals that emit a scent which, when perceived by dogs and humans, smell similarly to the illicit drug of interest [16, 17]. Meanwhile, they do not possess the health and addiction hazards of the drug. For example, a previous study by Sokolenko et al. reported the creation of scent mimics as training aids for drug detection dogs [18], with one motivation being to prevent the damage to the olfactory nerve endings and sense of smell of dogs caused by regular sniffing of authentic drugs such as cocaine. The authors developed and performed canine validation on a scent mimic for cocaine using methyl benzoate and methyl cinnamate, and one for amphetamine using propiophenone and benzaldehyde, or precursors to benzaldehyde which allow gradual and sustained release [18].

Indeed, the narcotic itself is often odourless, with the odour derived from by‐products of synthesis or decomposition products [19–23]. Previous studies, including those employing headspace gas chromatography–mass spectrometry (GC–MS) [21], revealed that the scent signature of cocaine is largely contributed by aromatic compounds including methyl benzoate, methyl cinnamate, and benzoic acid [20, 24–28], while the scent of methamphetamine is attributed to benzaldehyde, 1‐phenyl‐2‐propanone (P2P), *N*‐methylbenzylamine, and propiophenone [5, 6, 29–31]. In another report, GC–MS revealed the contributions of ethyl acetate and benzoic acid to the scent of cocaine, and of benzaldehyde, *N*‐methylbenzylamine and propiophenone to the scent of methamphetamine [32]. Based on these results, the author developed and performed canine validation on scent mimics of cocaine using benzoic acid as the single component, and of methamphetamine using propiophenone and benzaldehyde in a two‐component formulation [32].

Developing cost‐effective and extended‐range formulations that effectively recreate the distinctive odours of cocaine and methamphetamine, yet being safe for both canine and human use, would enable wider use of the training and instructional materials, benefiting especially the antidrug campaigns.

For public educational purposes, liquid‐form products may be impractical due to high costs, special storage requirements and safety concerns [17, 18, 21, 33]. Instead, an inert absorbent that converts these liquids into a solid scent‐carrier offers a safer and more feasible alternative. The choice of absorbent and carrier materials requires special attention. For example, some delivery systems employ silica gel or powdered materials such as diatomaceous earth, which can cause respiratory harm to dogs or humans on frequent exposure via silicosis [34, 35].

To effectively absorb the pseudoscent chemicals and allow for the gradual release of scent, porous materials are favourable [36]. One possible natural absorbent is cotton, in which cellulose fibres have demonstrated excellent sorption capacities for small organic drug molecules thanks to their high porosity [37]. Cotton also has high absorbency for polar species due to hydrogen bonding via its hydroxyl groups [38]. Among synthetic materials, metal‐organic frameworks (MOFs) offer porous structures with a balance between rigidity and flexibility [39–41]. MOFs have been reported to offer low‐cost and efficient extraction [42–44], detection [45, 46], and even removal of narcotics for treating acute overdoses [3]. While some prior works detailed the sorption and desorption of methamphetamine or related organic compounds between air and gauze materials [47–49], including for canine detection purposes [50, 51], a comparison of the effectiveness of cotton and MOF materials as pseudoscent carriers was yet to be investigated.

**FIGURE 1:**
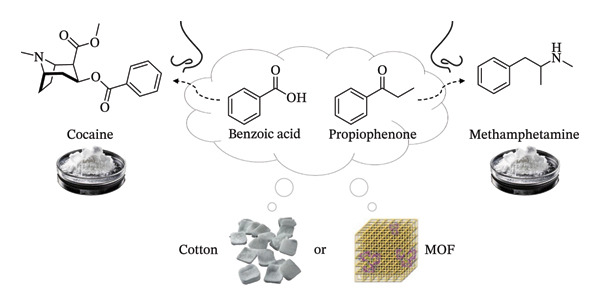
The pseudoscent delivery systems evaluated in this study and the composition substances, and the corresponding narcotic each of them represents.

Here, we considered a synthetic MOF and cotton as potential absorbent and carrier materials for formulating delivery systems for cocaine and methamphetamine pseudoscents, making use of benzoic acid and propiophenone, respectively (Figure 1). Given that these formulations are intended for human exposure, both the effectiveness and potential hazards of the pseudoscent chemicals were carefully considered. For cocaine pseudoscent, benzoic acid was validated as an effective single‐component scent mimic [32]. For methamphetamine pseudoscent, out of the reported scent components, P2P is a Schedule II‐controlled substance and thus avoided [52]. The safety aspect was considered by consulting substance information, including the Classification, Labelling and Packaging (CLP) Regulation on the European Chemicals Agency (ECHA) CHEM database. Whereas benzaldehyde (CAS Number 100‐52‐7) is associated with significant health risks such as potential damage to the unborn child and fertility [53], there is no such serious risk in using propiophenone (CAS Number 93‐55‐0), justifying a preference for its use for public educational purposes [54].

The effectiveness of absorption and the stability of storage were evaluated by a high‐performance liquid chromatography (HPLC)–based method. The cotton‐based delivery system exhibited superior absorption and storage abilities, as well as cost‐effectiveness, enabling dissemination of the material for canine training and public educational purposes.

## 2. Materials and Methods

### 2.1. Synthesis of MOF

The MOF material was synthesised (Supporting Scheme S1, Figures S1–S2) with reference to a previous report [39]. Briefly, 0.744 g of Zn(NO_3_)_2_·6H_2_O (Macklin Shanghai China, 98%) was dissolved in 4 mL of deionised water with stirring. 8.2 g of 2‐methylimidazole (2‐MeIM) (Macklin Shanghai China, 96%) was dissolved separately in 40 mL of deionised water. The solutions were separately incubated and homogenised in an ultrasonic bath for 5 min. Then, the two solutions were mixed by stirring at 500 rpm for 30 min at 20 °C. The resulting mixture was centrifuged at 6000 rpm for 30 min at 4 °C. The recovered product was washed three times with deionised water and lyophilised for 16 h to yield a dry powder.

### 2.2. Preparation of Pseudoscent Delivery Systems for Cocaine

To prepare the cotton‐based pseudoscent delivery system for cocaine, 1 g of benzoic acid (Energy Chemical, China, 99.5%) was dissolved in 100 mL of diethyl ether. The 100 mL solution was sprayed onto 100 g of cotton and left to dry at 20 °C under ambient air. The cotton was then stored in a closed glass jar for 14 days before subsequent analysis. The sealed glass jar was placed indoors on a laboratory bench in an air‐conditioned, window‐blinded room that was lamp‐lit during daytime, at 20 °C and at a relative humidity of 20%–30%. The MOF‐based delivery system was prepared similarly, but using 0.01 g of benzoic acid dissolved in 10 mL of diethyl ether, and 1 g of MOF.

### 2.3. Preparation of Pseudoscent Delivery Systems for Methamphetamine

**FIGURE 2:**
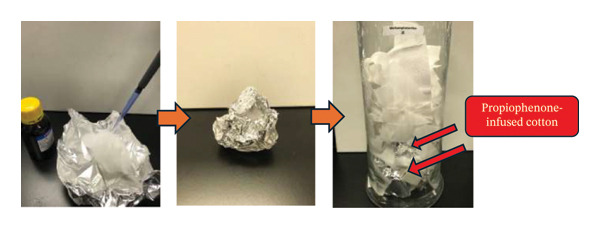
Preparation of the cotton‐based delivery system for the pseudoscent for methamphetamine using propiophenone.

For the cotton‐based pseudoscent delivery system for methamphetamine (Figure 2), 5 mL of propiophenone (Merck, 99.0%) was used to prepare propiophenone‐infused cotton loosely wrapped in aluminium foil. The foil‐wrapped propiophenone‐infused cotton was stored, together with 100 g of bulk cotton, in a closed glass jar for 14 days. The bulk cotton was loosely placed to allow for maximum and uniform absorption of evaporated propiophenone. The sealed glass jar was placed indoors on a laboratory bench in an air‐conditioned, window‐blinded room that was lamp‐lit during daytime, at 20 °C and at a relative humidity of 20%–30%. In subsequent analysis, samples were taken from the 100 g of bulk cotton.

The MOF‐based delivery system was prepared at a 10‐fold higher chemical‐to‐material ratio, albeit at a smaller scale (Figure S3). 0.5 mL of propiophenone was used to prepare propiophenone‐infused MOF loosely wrapped in aluminium foil. The foil‐wrapped propiophenone‐infused MOF was stored with 1 g of bulk MOF in a closed glass vial for 14 days. The bulk MOF was dispersed to allow for maximum and uniform absorption of evaporated propiophenone. The closed glass vial was placed indoors on a laboratory bench in an air‐conditioned, window‐blinded room that was lamp‐lit during daytime, at 20 °C and at a relative humidity of 20%–30%. In subsequent analysis, samples were taken from the 1 g of bulk MOF.

With a density of 1.009 g mL^−1^, if all propiophenone were fully transferred to the bulk absorbent material, the full absolute loadings of propiophenone on the cotton and MOF materials would be 50 and 500 mg/g, respectively.

### 2.4. HPLC

Analysis of pseudoscent substances was performed using HPLC with diode‐array detection (HPLC–DAD), using an Agilent 1100 HPLC system. Conditions were as follows: ZORBAX Eclipse XDB C‐18 4.6 × 150 mm 5 μm column; column at room temperature; injection volume of 20 μL; flow rate of 1 mL min^−1^; total run time of 15 min; solvent gradient of 30% to 90% acetonitrile in water from *t* = 3 to 15 min. The same detection wavelength of 254 nm was applied in the detection of both benzoic acid and propiophenone.

### 2.5. Determination of Pseudoscent Contents in Carrier Materials

Standard solutions at five different concentrations (5, 10, 20, 25 and 50 ppm for benzoic acid; 2.5, 10, 20, 25 and 50 ppm for propiophenone) were prepared in acetonitrile. Each standard solution was analysed by HPLC in triplicate (see above run conditions). Calibration curves were obtained by plotting the mean peak areas against analyte concentrations using linear regression on Microsoft Excel.

Pseudoscent carriers were analysed 14 days following preparation (Figures S4‐S5). For the cotton‐based pseudoscent carriers, they were cut and divided into 1.0 g samples. For the MOF‐based carriers, 0.1‐g samples were weighed. Each sample was placed in a 20 mL extraction vial. To each vial, acetonitrile (10 mL for the 1.0‐g cotton samples; 1 mL for the 0.1‐g MOF samples) was added as the extraction solvent, and the mixture was sonicated using an ultrasonicator (Crest Ultrasonics 2600D) at 45 kHz at a peak/effective power of 375/187.5 W for 30 min in a water bath at 20 °C. 1 mL of the supernatant was filtered through a 0.22‐μm filter, diluted by a factor of 50 or 20 for benzoic acid or propiophenone samples, respectively, before being transferred into a vial for HPLC analysis (see above). Results (concentrations in mg g^−1^) were reported as average values (± standard deviation, SD) across five independent sample replicates (*n* = 5).

### 2.6. Method Validation of HPLC‐Based Pseudoscent Content Analysis

Method accuracy was evaluated by spike‐and‐recovery experiments. For each analyte‐carrier combination, three spiking levels were considered. Known analyte concentrations were spiked in the cotton‐based (1 g) or MOF‐based (0.1 g) samples, before extraction and HPLC analysis as described above. Recovery (%) was then calculated as: (Observed concentration − Endogenous concentration)/Spiked diluent concentration × 100%. For each analyte‐carrier combination, the experiment was repeated at least three times.

Method precision was assessed by evaluating the relative standard deviation (RSD) from repeated analyses of samples at the same spiked concentration.

Method sensitivity was evaluated by the limits of detection (LOD) and quantification (LOQ). Samples with known low analyte concentrations were analysed, and the concentrations at which the signal‐to‐noise ratio (S/N) equals 3 and 10 correspond to the LOD and LOQ, respectively.

### 2.7. Analysis of Volatile Emission Profiles Over Time

Pseudoscent delivery systems (0.1 g cotton or MOF) were prepared for time‐course analysis (Figures S6‐S8). For each material–analyte combination, five samples were prepared in sealed 20‐mL vials for incubation over 0, 1, 3, 7 and 14 days, on a laboratory bench in an air‐conditioned, window‐blinded room that was lamp‐lit during daytime, at 20 °C and at a relative humidity of 20%–30%. For benzoic acid systems, the benzoic acid/diethyl ether solution as described in ‘Preparation of Pseudoscent Delivery Systems for Cocaine’ above was added directly onto the cotton or MOF material, and the solvent allowed to evaporate completely in ambient air prior to sealing the vial. For the propiophenone–cotton system, propiophenone was added directly and allowed to evaporate in ambient air prior to sealing the vial. For the propiophenone–MOF system, a vial containing 0.1 g of MOF was situated within a larger sealed container containing a reservoir of propiophenone, in a setup similar to that described in ‘Preparation of Pseudoscent Delivery Systems for Methamphetamine’ above. The MOF was loaded via passive vapour diffusion until equilibrium was reached, after which it was transferred to a 20‐mL vial and sealed.

Headspace volatile analyte content in the vial was determined using GC–MS analysis, using an Agilent 7890A Gas Chromatograph coupled to a 5975C Mass Spectrometer. To obtain the headspace sample, the vial septum was pierced with a gas‐tight syringe, and a 100‐μL aliquot was withdrawn for GC–MS analysis. Separation was achieved on an HP‐5MS Ultra Inert column (30 m × 0.25 mm, 0.25 μm film thickness). The GC oven temperature program initiated at 50 °C, followed by a ramp to 180 °C over a total run time of 10 min. MS detection was performed in selected ion monitoring (SIM) mode to ensure high sensitivity and selectivity for benzoic acid and propiophenone. Peak areas were integrated to represent the relative headspace concentration. Negative controls, consisting of cotton‐only or MOF‐only vials, were analysed to confirm the absence of interferences.

Following GC–MS analysis, the analyte content retained on the cotton or MOF material was determined using the extraction and HPLC–DAD procedures described in ‘Determination of Pseudoscent Contents in Carrier Materials’ above.

### 2.8. Powder X‐Ray Diffraction (XRD)

Powder XRD patterns of unloaded MOF, benzoic acid‐loaded MOF and propiophenone‐loaded MOF were recorded using a PANalytical Empyrean diffractometer with a Cu Kα anode (*λ* = 0.15406 nm) at 40 kV and 40 mA.

### 2.9. Thermogravimetric Analysis (TGA)

TGA was performed using a Perkin Elmer TGA‐6 Thermal Gravimetric Analyzer. Each of the MOF samples (10–12 mg) or activated carbon samples (2–5 mg) was heated from 47 °C to above 500 °C, with the percent weight retained monitored.

### 2.10. Analysis of Headspace Volatile Organic Compound (VOC) Content

To assess headspace vapour concentrations, a portable VOC meter (Pulitong portable composite gas analyser, PLT300‐VOC), with a manufacturer‐specified LOD of 1.0 ppm, was utilised. Sampling of headspace was conducted either at the opening of an opened 20‐mL vial containing 0.1 g of pseudoscent‐infused cotton or at the opening of the benzoic acid or propiophenone reagent bottle. The measured VOC content in units of ppm was converted to an estimated concentration (mg m^−3^) by multiplying the reading (ppm) by the molecular weight (g mol^−1^) and dividing by the molar volume of gas at 1 atm and 25 °C (24.5 dm^3^ mol^−1^).

## 3. Results and Discussion

### 3.1. Characterisation of MOF Products

The preparation of the cotton‐based and MOF‐based delivery systems for benzoic acid and propiophenone, which, respectively, mimic the scents of cocaine and methamphetamine, was described (Figure 2; Figures S1‐S3). In total, four types of pseudoscent delivery systems were prepared. In all cases, the formulation was simple, involving minimal preparation and, if any, synthesis steps. The materials and chemicals used were inexpensive and readily available. These will enable easy access by government and law enforcement agencies to the canine training and educational materials, in quantities suitable for conducting exhibitions and workshops in various community settings.

The pseudoscent loading of MOF was further characterised. In the final step of the synthesis of the zeolitic imidazolate framework (ZIF) MOF, the product was washed with deionised water, followed by a 16‐h lyophilisation, thus activating the ZIF material for uptake of molecules. To check the crystallinity of ZIF before and after loading, powder XRD was performed (Figure S9). The sharp, well‐defined peaks in the powder XRD patterns confirmed the crystallinity of ZIF both before and after loading with benzoic acid and propiophenone. However, compared to the unloaded ZIF, loading of both benzoic acid and propiophenone resulted in the lowest‐angle peak shifting to higher angles, reflecting a contraction in lattice size following loading. In the case of propiophenone, an overlay of peaks was observed, possibly reflecting defects in part of the structure.

Loading was further characterised by TGA (Figure S10). For comparison, activated carbon systems to which benzoic acid or propiophenone was adsorbed were also characterised. Comparing the TGA profiles of the unloaded and loaded MOFs, a 5%–8% uptake by weight of the pseudoscent chemicals in MOF was indicated by the drop in percent weight of the loaded MOFs around 120 °C–150 °C, with decomposition of MOF occurring at higher temperatures. By contrast, activated carbon, to which pseudoscent chemicals were adsorbed, was not a suitable delivery agent for pseudoscents, as evidenced by the lack of significant chemical release until much higher temperatures than the MOF systems.

### 3.2. Method Validation

**FIGURE 3:**
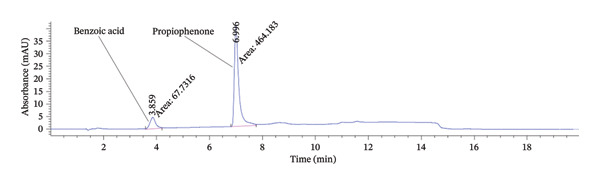
Chromatogram of a mixed 10 ppm standard of benzoic acid and propiophenone, with peak areas in units of mAU·s.

**FIGURE 4:**
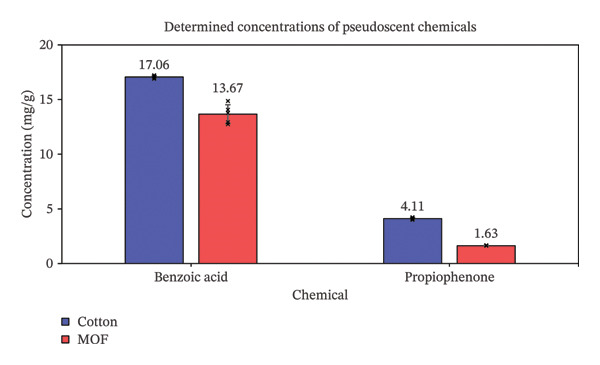
Determined concentrations (± standard deviation, SD; *N* = 5) of pseudoscent chemicals in different carrier materials.

The benzoic acid and propiophenone contents in the absorbent materials were quantitatively determined, via sonication‐assisted extraction with acetonitrile (Figures S4‐S5), followed by HPLC–DAD analysis of the resulting extract. Overall, HPLC enabled excellent separation, with benzoic acid showing a retention time of *t* = 3.70–4.10 min, and propiophenone *t* = 6.80–7.10 min, as expected considering the higher polarity of benzoic acid (Figure 3). Analyte quantification was achieved by calibration curves which displayed high linearity (*R* ^2^ ≥ 0.9999) within the concentration ranges of interest (Figure 4; Figures S11‐S12; Tables S1‐S3). The identities of analyte peaks were confirmed by spiking (Tables S4‐S7).

According to the International Council for Harmonisation of Technical Requirements for Pharmaceuticals for Human Use (ICH) guidelines on validation of analytical procedures, the accuracy of an analytical method should be assessed using spike‐and‐recovery experiments at a minimum of three concentration levels with a minimum of nine determinations in total [55]. Hence, for each of the four systems, accuracy of the extraction and analysis protocol was assessed using spike‐and‐recovery experiments at the low (2.5–10 ppm), medium (20–25 ppm), and high (40–50 ppm) concentration levels, at each of which at least triplicate measurements were performed. The acceptable range is typically 80%–120% recovery. For cotton‐based systems, the recoveries for benzoic acid and propiophenone were 101%–118% and 94%–103%, respectively (Tables S4‐S5). For MOF‐based systems, the recoveries for benzoic acid and propiophenone were 92%–109% and 94%–109%, respectively (Tables S6‐S7). All the values were closely clustered around 100%, indicating that the extraction method was successful in extracting the chemicals of interest from cotton and MOF. The HPLC‐based analysis also achieved high precision, with repeated analyses of spiked samples yielding RSD values within 4% (Table S8). Finally, the LOD and LOQ were determined as the analyte concentrations at which the S/Ns equal 3 and 10, respectively. For benzoic acid and propiophenone, the LODs were 0.131 and 0.100 ppm, respectively; the LOQs were 0.435 and 0.333 ppm, respectively (Table S8). The sub‐ppm values suggested that the method is sufficiently sensitive, and demonstrated comparable sensitivity to previously reported LOD values for liquid chromatography‐based detection of benzoic acid (0.2 ppm and 0.404 ppm) [56, 57] and of propiophenone (0.14 ppm) [58].

The excellent linearity of the calibration curves (*R* ^2^ ≥ 0.9999) for benzoic acid (5–50 ppm) and propiophenone (2.5–50 ppm) was verified via analysis of back‐calculated standards (Tables S9‐S11). The extraction procedure demonstrated itself to be free from significant matrix effects through post‐extraction addition experiments (Tables S12‐S13), and robust against minor variations in extraction time and solvent volume (Tables S14‐S15), with carryover being absent in blank injections into the HPLC–DAD system. Overall, the extraction and HPLC‐based quantification methods were fully validated to ensure high quality, robustness and reliability of subsequent analysis.

### 3.3. HPLC‐Based Determination of Analyte Content in Cotton and MOF Carriers

Using the validated method of extraction and analysis, the concentrations of pseudoscent chemicals in each carrier material were determined (Figure 4), 14 days following preparation. The time interval was selected based on the envisaged use of the delivery systems in canine training and educational exhibitions or workshops. A fortnightly preparation of fresh materials was reasonable in terms of preparation cost and effort, which, as described, was minimal, and in terms of the durability and stability of the scents. Comparing the two carrier materials (Figure 4), for benzoic acid, the concentration in MOF was (13.67 ± 0.86) mg/g, which was different (*p* < 0.01) and lower than that in cotton at (17.06 ± 0.13) mg/g. For propiophenone, even though the MOF‐based system has a 10‐fold higher propiophenone‐to‐material ratio than the cotton‐based system, the concentration of propiophenone in MOF at (1.63 ± 0.03) mg/g was different (*p* < 0.01) and lower than that in cotton at (4.11 ± 0.10 mg/g). If the absolute loading ratios were to be the same at 50 mg propiophenone per g of absorbent material, as per the cotton‐based system, the expected concentration of propiophenone in MOF at approximately 0.16 mg/g would be much lower than that in cotton at 4.11 mg/g. Given the demonstrated excellent recoveries, these data indicated that smaller amounts of pseudoscent chemicals were present in the MOF‐based delivery systems than the cotton‐based ones at the time of analysis. The chemicals were not as effectively transferred into the pores of the MOF, compared to cotton, with the difference being more significant for propiophenone, which had to evaporate from the infused material before capture by the bulk material, both processes being potentially less efficient for MOF than cotton. The detectable amounts of analytes in the cotton material suggested that cotton can act as an inert carrier for the pseudoscent chemicals, which remained present and stable for at least two weeks following preparation. Cotton is the superior absorbent material for carrying the scent mimic in detectable amounts for a prolonged period.

### 3.4. Headspace and Retained Analyte Content in Cotton and MOF Carriers Over Time

While the previous analysis indicated cotton to be the superior absorbent material for retaining pseudoscent chemicals, the utility of the pseudoscent carrier in canine training and public education relies on odour emission and thus the presence of pseudoscent vapour in the headspace above the material. To study headspace concentration and temporal release of pseudoscents, volatile emission profiles from cotton and MOF carriers, each of which carries benzoic acid or propiophenone, were constructed using GC–MS (Tables S16‐S17 and Figures S13‐S16). Both analytes demonstrated a sustained release into the headspace over the 14‐day period. For each chemical, headspace concentration was lower for MOF than for cotton at equivalent time points, indicating that the lower analyte content retained in MOF than in cotton after 14 days was due to poorer absorption during initial loading, rather than faster evaporative loss. In all systems except propiophenone‐infused MOF, headspace concentration plateaued to equilibrium after storage for 14 days, thereby justifying the 14‐day storage period in the preparation of pseudoscent delivery systems.

The stability profile of pseudoscent chemicals on the cotton and MOF materials was characterised by quantifying the analyte content remaining on the materials, using the described extraction and HPLC‐based determination procedures (Tables S18‐S19). Overall, the retained concentration of benzoic acid and propiophenone remained stable and within ±10% of the initial loading on Day 0. This confirmed the absence of any significant losses or chemical degradation of the pseudoscent chemicals on the solid carrier materials over the time period considered. Considering the superior initial uptake, sustained headspace concentration, and stability profiles (Table S20), the choice of cotton in scent‐mimic delivery systems over MOF is justified.

### 3.5. Safety and Utilisation

Finally, the scent‐mimic delivery systems need to be safe for educational purposes. Cotton pads are commonplace household items which have minimal hazards, and given that they are placed in a container, such as a glass jar or sealable bag, participants’ contact with them should be minimal. To acquaint participants with the scents mimicking cocaine or propiophenone, it is recommended that about 0.1 g of pseudoscent‐infused cotton be placed in a 20‐mL capped glass vial, briefly opened for exposure, or scaled up to 0.5 g of cotton in a glass jar or sealable bag of 100 mL capacity. Participants are usually exposed for 2–5 s, with a maximum of 5 s to prevent overexposure. After the educational activity, the used materials are placed in sealed bags and disposed of as standard refuse.

In terms of the hazards of the pseudoscent chemicals themselves, substance information including the CLP Regulation was consulted on the ECHA CHEM database. According to the CLP harmonised classification and labelling, propiophenone (CAS Number 93‐55‐0) has no associated hazards [54]. On the other hand, benzoic acid (CAS Number 65‐85‐0) is classified to cause skin irritation, serious eye damage, and damage to the lungs through prolonged or repeated exposure if inhaled [59]. To assess the hazard of pseudoscent exposure, the headspace VOC concentration was estimated using a portable VOC meter (LOD = 1.0 ppm). Placing the VOC meter at the opening of the reagent bottles of benzoic acid and propiophenone resulted in readings of 2 ppm (10 mg m^−3^) and 70–80 ppm (380–440 mg m^−3^), respectively, reflecting the higher volatility of propiophenone compared to benzoic acid. By contrast, the reading remained at 0.0 ppm for the vial headspace above the cotton material infused with either benzoic acid or propiophenone. Considering the LOD of 1.0 ppm, the headspace concentrations of benzoic acid and propiophenone from cotton carriers were estimated to be < 5.0 mg m^−3^ and < 5.5 mg m^−3^, respectively. For the more hazardous benzoic acid, the headspace concentration of < 5.0 mg m^−3^ was below the time‐weighted average of 10 mg m^−3^ in the EH40/2005(GB) inhalable occupational exposure limit [60]. Considering the exposure time to be within 5 s, the health impact from inhalation of benzoic acid is minimal.

Therefore, with the containment of cotton‐based pseudoscent carriers in educational workshop settings, direct contact by participants is unlikely. Considering the effective absorption and retention of benzoic acid by cotton, and the short duration of inhalation by participants, the health hazards to dogs and humans are considered minimal, especially when compared to using authentic cocaine samples.

## 4. Conclusions

We present easily formulated, stored, and distributed delivery systems for scent mimics of cocaine and methamphetamine, using benzoic acid and propiophenone, respectively, with cotton shown to be the superior inert absorbent and carrier material. The delivery systems are safe, durable and inexpensive, thus exhibiting realistic potential for widespread use in service dog training and anti‐narcotic public education. Through such interactive olfactory experiences in workshops and exhibitions, the unpleasant scents not only provide a deterrent to illicit drug use, but also help educate the public to detect potential drug abuse early, especially among young individuals. Indeed, the cotton‐based cocaine and methamphetamine scent‐mimic formulations have been utilised to enhance engagement in public campaigns and deliver anti‐drug messages, the effectiveness of which has been evaluated by Lai et al. [15].

While the analysis indicated that cotton is a better inert absorbent than the synthesised MOF, we recognise possibilities of further optimisation of MOF properties, including the control of pore sizes by variations in the metal or ligand and by the synthesis conditions [39]. For example, microfluidic gradient mixing of the metal, ligand and pseudoscent chemicals may result in a pseudoscent–MOF composite that enables gradual release of the pseudoscent molecules, with those near the surface or in larger pores being released first [39]. The potential of small molecule–MOF composites in the encapsulation and controlled release of aroma molecules is of interest with wide‐ranging applications [41].

## Funding

This work was supported by Hong Kong Baptist University Seed Fund to Catherine H. H. Hor and Kangning Ren.

## Conflicts of Interest

The authors declare no conflicts of interest.

## Supporting Information

The Supporting Information file includes the following.

Scheme S1: Synthesis of MOF from Zn(NO_3_)_2_·6H_2_O and 2‐methylimidazole (2‐MeIM).

Figure S1: Synthesis of MOF.

Figure S2: MOF product as a dry product after lyophilisation.

Figure S3: Preparation of the MOF‐based pseudoscent delivery system for methamphetamine using propiophenone.

Figure S4: Sample preparation for the analysis of cotton‐based pseudoscent delivery systems.

Figure S5: Sample preparation for the analysis of MOF‐based pseudoscent delivery systems.

Figure S6: Preparation of benzoic acid‐ or propiophenone‐infused cotton for GC–MS analysis.

Figure S7: Preparation of benzoic acid‐infused MOF for GC–MS analysis.

Figure S8: Preparation of propiophenone‐infused MOF for GC–MS analysis.

Figure S9: Powder X‐ray diffraction (XRD) patterns of unloaded MOF, and MOF loaded with benzoic acid (BA) and propiophenone (PP).

Figure S10: Thermogravimetric analysis (TGA) curves of unloaded and loaded MOFs, and benzoic acid‐ or propiophenone‐loaded activated carbon systems.

Figure S11: Calibration curve for benzoic acid.

Figure S12: Calibration curve for propiophenone.

Table S1: Determined concentrations of benzoic acid (pseudoscent chemical for cocaine) in different carrier materials, using five independent sample replicates (*n* = 5).

Table S2: Determined concentrations of propiophenone (pseudoscent chemical for methamphetamine) in different carrier materials, using five independent sample replicates (*n* = 5).

Table S3: Determined concentrations (± standard deviation, SD; *n* = 5) of pseudoscent chemicals in different carrier materials.

Table S4: Recovery of benzoic acid from the cotton‐based delivery system.

Table S5: Recovery of propiophenone from the cotton‐based delivery system.

Table S6: Recovery of benzoic acid from the MOF‐based delivery system.

Table S7: Recovery of propiophenone from the MOF‐based delivery system.

Table S8: Measured chromatogram peak areas in the repeated analyses of samples, and precision and sensitivity parameters, in the determination of benzoic acid and propiophenone.

Table S9: Back‐calculation for calibration curve of benzoic acid.

Table S10: Back‐calculation for calibration curve of propiophenone.

Table S11: Verification using triplicate QC samples prepared at 10 ppm.

Table S12: Recovery from post‐extraction spike for cotton.

Table S13: Recovery from post‐extraction spike for MOF.

Table S14: Robustness tests for cotton samples.

Table S15: Robustness tests for MOF samples.

Table S16: Headspace analyte vapour content over pseudoscent‐infused cotton by GC–MS.

Table S17: Headspace analyte vapour content over pseudoscent‐infused MOF by GC–MS.

Figure S13: Headspace concentration profile for benzoic acid‐infused cotton.

Figure S14: Headspace concentration profile for propiophenone‐infused cotton.

Figure S15: Headspace concentration profile for benzoic acid‐infused MOF.

Figure S16: Headspace concentration profile for propiophenone‐infused MOF.

Table S18: Retained benzoic acid content in material by HPLC.

Table S19: Retained propiophenone content in material by HPLC.

Table S20: Mass stability of cotton‐based pseudoscent delivery systems.

## Supporting information


**Supporting Information** Additional supporting information can be found online in the Supporting Information section.

## Data Availability

The data that support the findings of this study are available in the supporting information of this article.
