# Supplementary material for: Formulation and Delivery Systems of Cocaine and Methamphetamine Scent Mimics
Source: Int J Anal Chem. 2026 Feb 4;2026:2222059. doi: 10.1155/ianc/2222059 (PMC12872962; doi:10.1155/ianc/2222059)
Supplement: Supplementary file 1 — Supporting Information Additional supporting information can be found online in the Supporting Information section. [file IANC-2026-2222059-s001.pdf]

Supplementary Information to

Formulation and Delivery Systems of Cocaine and Methamphetamine Scent Mimics

Silvia T Mo,[#1] Issac C C Cheng,[#1] H T Henry Chan,[#1] Kin Yat Tong,[1] Zhengpei Li,[1] Yang Liu,[1] Kelvin S Y Leung,[1] Kangning Ren*[1] and Catherine H H Hor*[1]

[1] Department of Chemistry, Hong Kong Baptist University, Hong Kong S.A.R., China

[#] First authors equal

* Corresponding authors: kangningren@hkbu.edu.hk; catherinehor@hkbu.edu.hk

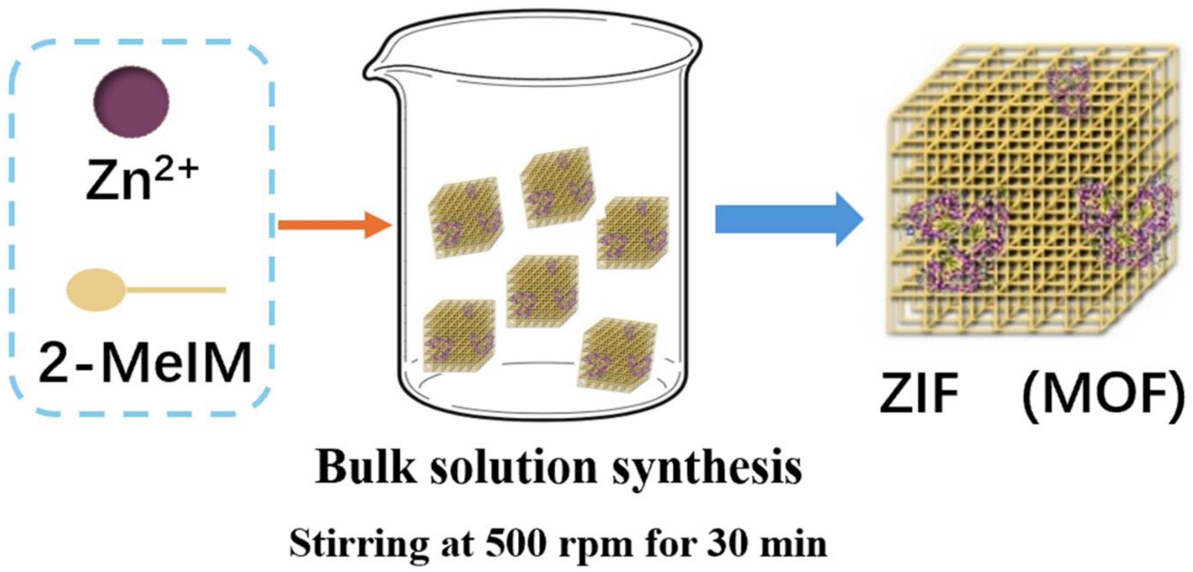

Scheme S1: Synthesis of MOF from $Zn(NO_3)_2 \cdot 6H_2O$ and 2-methylimidazole (2-MeIM).

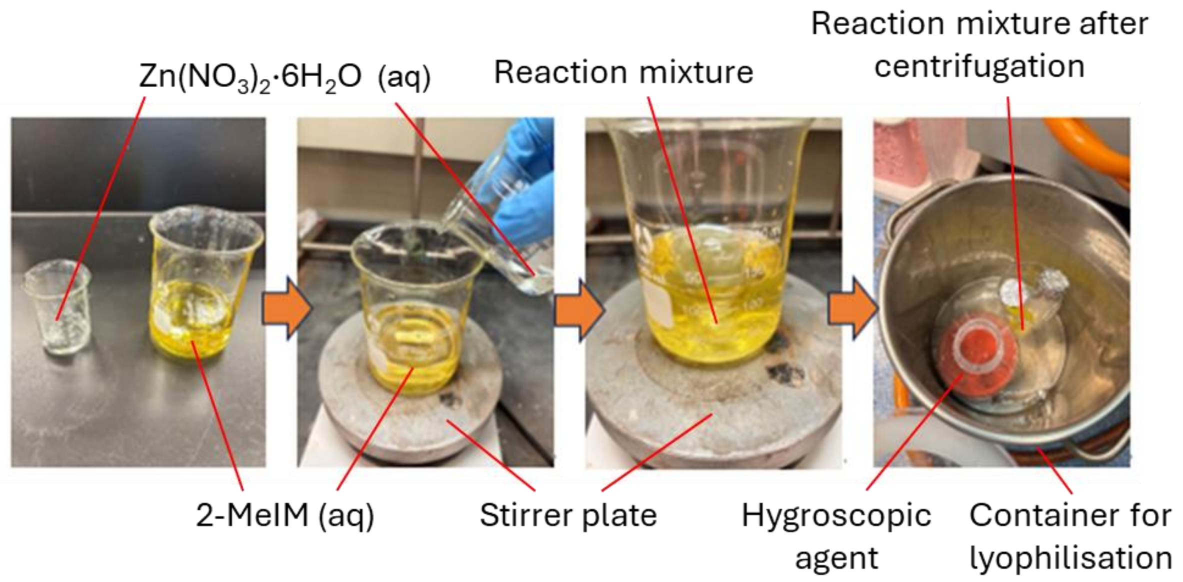

Fig. S1: Synthesis of MOF.

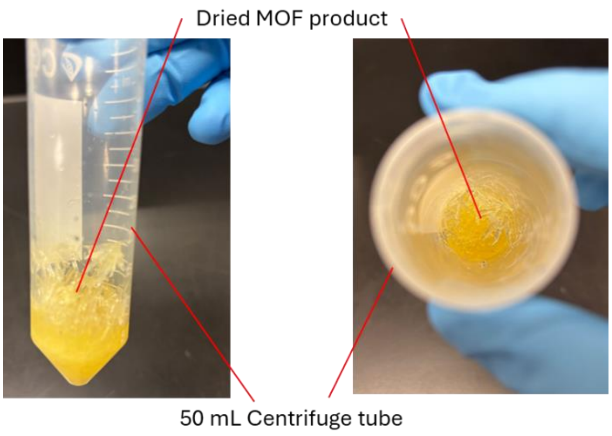

Fig. S2: MOF product as dry product after lyophilisation.

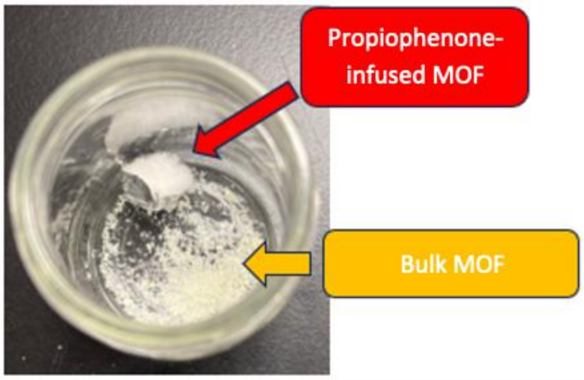

Fig. S3: Preparation of the MOF-based pseudoscent delivery system for methamphetamine using propiophenone.

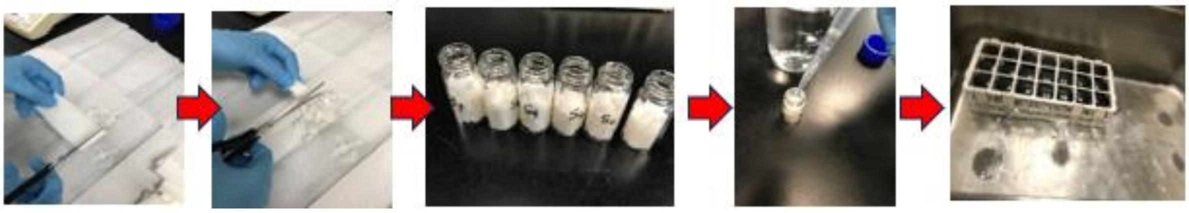

Fig. S4: Sample preparation for the analysis of cotton-based pseudoscent delivery systems.

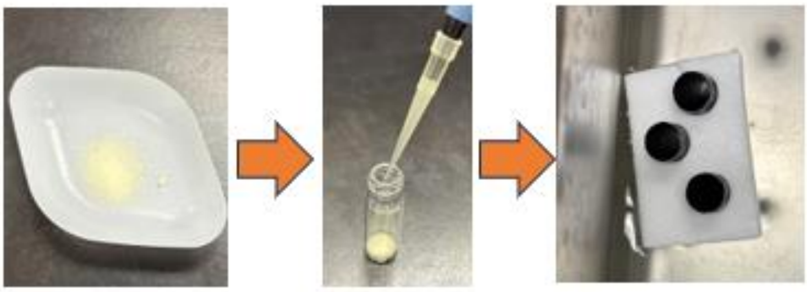

Fig. S5: Sample preparation for the analysis of MOF-based pseudoscent delivery systems.

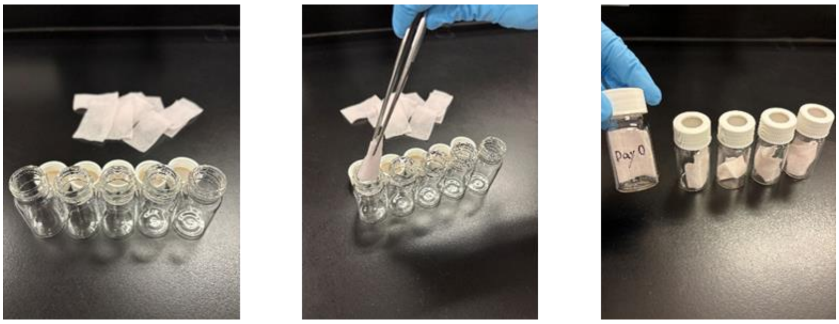

Fig. S6: Preparation of benzoic acid- or propiophenone-infused cotton for GC-MS analysis.

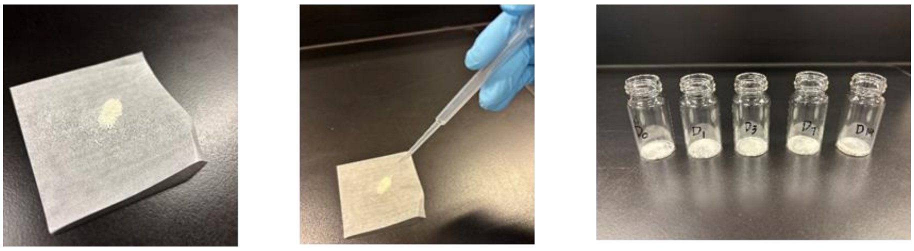

Fig. S7: Preparation of benzoic acid-infused MOF for GC-MS analysis.

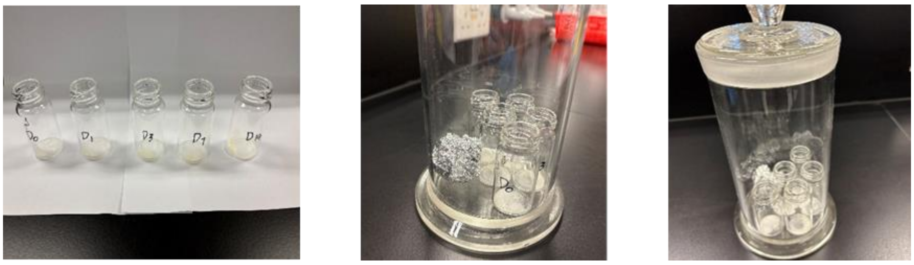

Fig. S8: Preparation of propiophenone-infused MOF for GC-MS analysis.

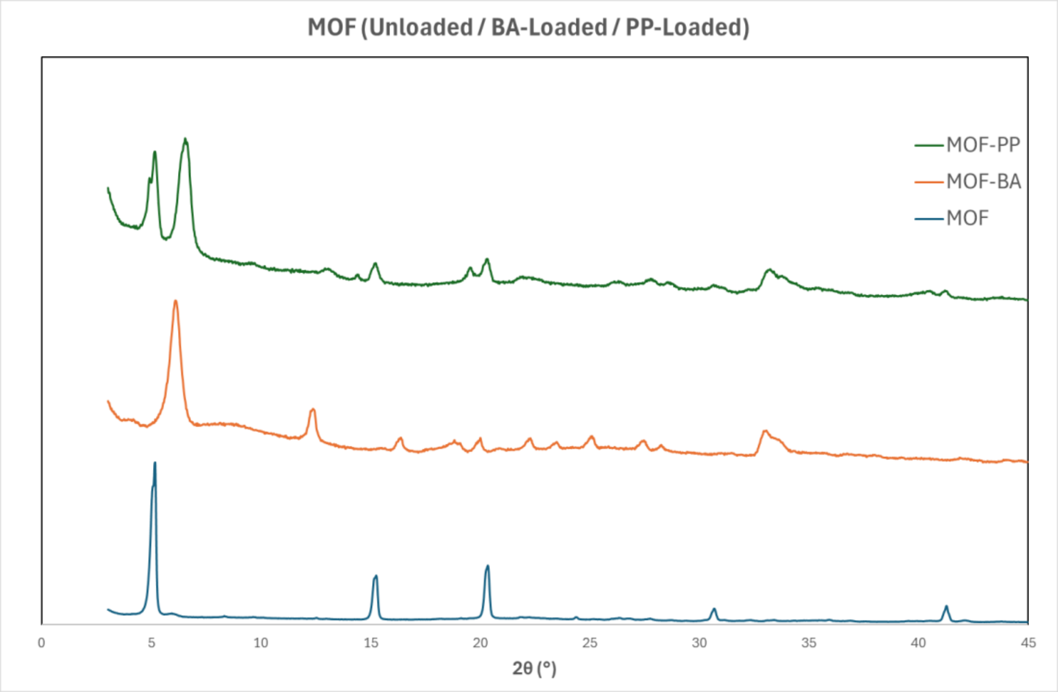

Fig. S9: Powder X-ray diffraction (XRD) patterns of unloaded MOF, and MOF loaded with benzoic acid (BA) and propiophenone (PP).

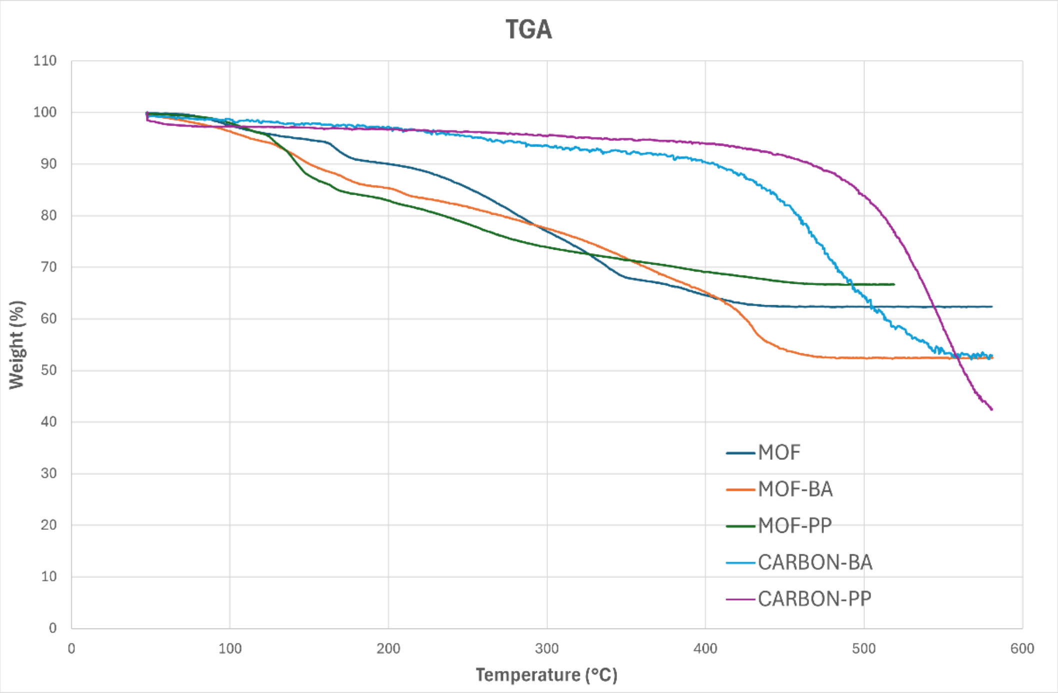

Fig. S10: Thermogravimetric analysis (TGA) curves of unloaded and loaded MOFs, and benzoic acid- or propiophenone-loaded activated carbon systems.

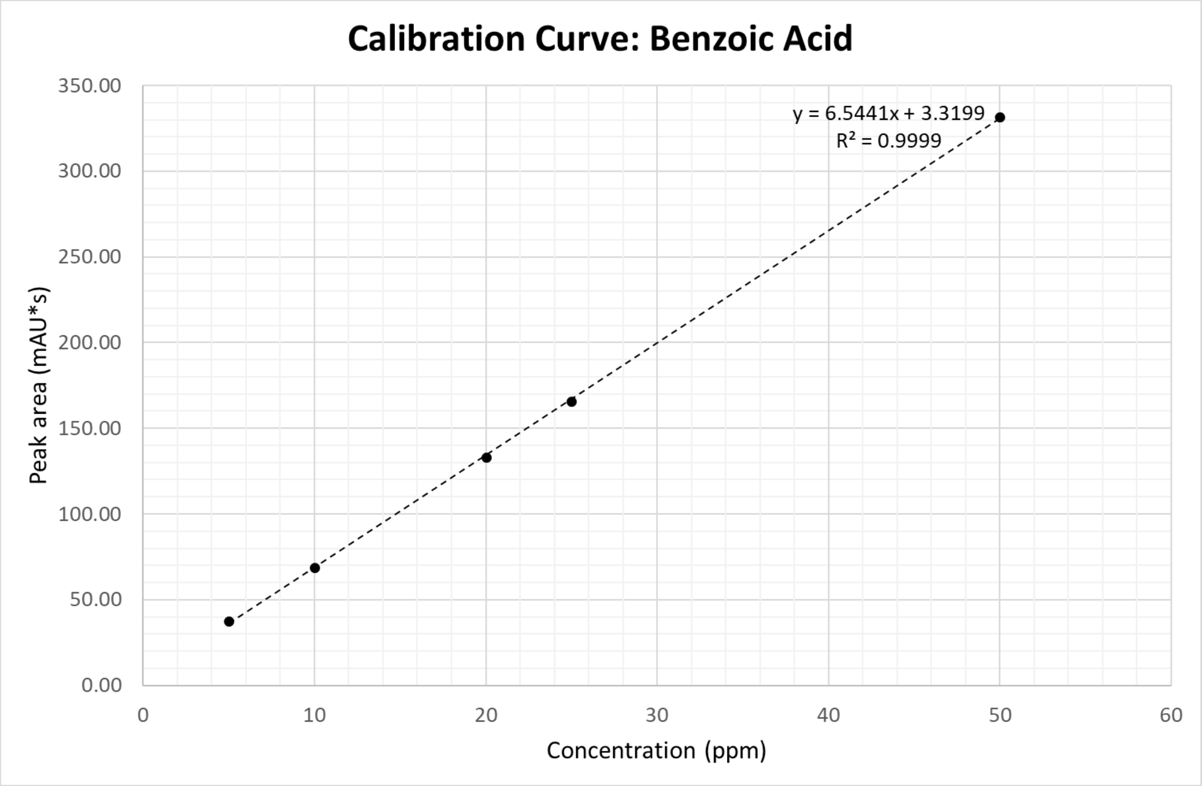

Fig. S11: Calibration curve for benzoic acid.

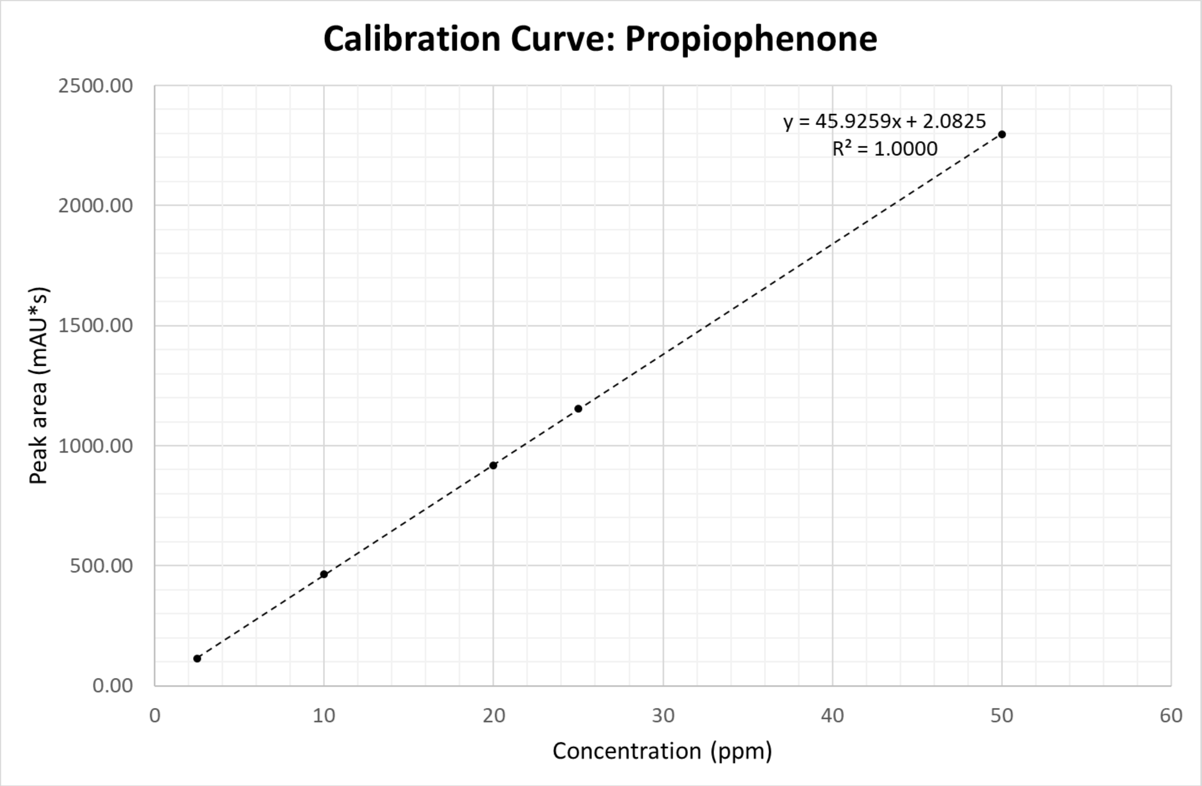

Fig. S12: Calibration curve for propiophenone.

Table S1: Determined concentrations of benzoic acid (pseudoscent chemical for cocaine) in different carrier materials, using five independent sample replicates (n = 5).

	Concentration (mg/g) – Benzoic acid	
	Material	
Trial	Cotton[a]	MOF[b]
1	17.08	14.86
2	16.88	13.76
3	17.13	12.93
4	17.22	12.74
5	16.98	14.06
Average	17.06	13.67
95% Confidence Interval	16.89 – 17.22	12.60 – 14.74
Two-tailed t-test: p-value	2.450×10^{-5}	

[a]: 1.0 g sample was analysed after extraction with 10 mL acetonitrile.

[b]: 0.1 g sample was analysed after extraction with 1 mL acetonitrile.

Table S2: Determined concentrations of propiophenone (pseudoscent chemical for methamphetamine) in different carrier materials, using five independent sample replicates (n = 5).

	Concentration (mg/g) – Propiophenone	
	Material	
Trial	Cotton[a]	MOF[b]
1	4.23	1.65
2	4.10	1.67
3	3.98	1.61
4	4.19	1.61
5	4.05	1.61
Average	4.11	1.63
95% Confidence Interval	3.98 – 4.24	1.59 – 1.67
Two-tailed t-test: p-value	1.92×10^{-11}	

[a]: 1.0 g sample was analysed after extraction with 10 mL acetonitrile.

[b]: 0.1 g sample was analysed after extraction with 1 mL acetonitrile.

Table S3: Determined concentrations (± standard deviation, SD; n = 5) of pseudoscent chemicals in different carrier materials.

Concentration (mg/g)	Material	
	Cotton	MOF
Benzoic acid	17.06 ± 0.13	13.67 ± 0.86
Propiophenone	4.11 ± 0.10	1.63 ± 0.03

Table S4: Recovery of benzoic acid from the cotton-based delivery system.

			Benzoic acid	
Spiking level	Spiked conc. (ppm)	Trial	Conc. in cotton (ppm)	Recovery (%)
Low	5	1	5.1622	103.24
		2	5.7605	115.21
		3	5.8920	117.84
		4	5.7673	115.35
		5	5.8173	116.35
		6	5.5501	111.00
Medium	25	1	28.9409	115.76
		2	27.7024	110.81
		3	28.5870	114.35
High	50	1	50.5419	101.08
		2	58.0625	116.13
		3	57.2291	114.46

Table S5: Recovery of propiophenone from the cotton-based delivery system.

			Propiophenone	
Spiking level	Spiked conc. (ppm)	Trial	Conc. in cotton (ppm)	Recovery (%)
Low	2.5	1	2.5434	101.74
		2	2.5629	102.51
		3	2.3646	94.58
		4	2.4689	98.76
		5	2.3714	94.86
		6	2.4854	99.42
Medium	25	1	24.5758	98.30
		2	24.5192	98.08
		3	24.4506	97.80
High	50	1	48.6988	97.40
		2	50.9882	101.98
		3	49.6525	99.31

Table S6: Recovery of benzoic acid from the MOF-based delivery system.

			Benzoic acid	
Spiking level	Spiked conc. (ppm)	Trial	Conc. in MOF (ppm)	Recovery (%)
Low	10	1	9.6235	96.24
		2	9.6473	96.47
		3	9.5523	95.52
		4	10.3242	103.24
		5	10.7042	107.04
		6	10.0511	100.51
Medium	20	1	19.5159	97.58
		2	21.1191	105.60
		3	19.0290	95.14
		4	20.5609	102.80
		5	19.3971	96.99
		6	18.5302	92.65
High	40	1	38.3742	95.94
		2	41.0937	102.73
		3	40.2743	100.69
		4	39.8587	99.65
		5	43.5757	108.94
		6	41.1412	102.85

Table S7: Recovery of propiophenone from the MOF-based delivery system.

			Propiophenone	
Spiking level	Spiked conc. (ppm)	Trial	Conc. in MOF (ppm)	Recovery (%)
Low	10	1	9.8441	98.44
		2	9.4548	94.55
		3	10.3382	103.38
		4	10.8255	108.25
		5	10.3223	103.22
		6	10.3132	103.13
Medium	20	1	19.8693	99.35
		2	21.2900	106.45
		3	20.5751	102.88
		4	19.5391	97.70
		5	20.4134	102.07
		6	20.5250	102.63
High	40	1	37.9978	94.99
		2	40.5798	101.45
		3	38.1231	95.31
		4	40.5434	101.36
		5	40.5707	101.43
		6	42.8772	107.19

Table S8: Measured chromatogram peak areas in the repeated analyses of samples, and precision and sensitivity parameters, in the determination of benzoic acid and propiophenone.

		Benzoic acid	Propiophenone
Spiked concentration		5 ppm	2.5 ppm
Peak area (mAU s) in Trial	1	37.1016	118.8900
	2	41.0173	119.7840
	3	41.8775	110.6790
	4	41.0620	115.4710
	5	41.3886	110.9920
	6	39.6403	116.2280
Average peak area (mAU s)		40.35	115.34
SD (mAU s)		1.76	3.84
RSD		4%	3%
LOD (ppm)		0.131	0.100
LOQ (ppm)		0.435	0.333

Back-calculated standards

The accuracy of the curves was confirmed by back-calculating the concentration of each standard, with an acceptance criterion of ±10% deviation from the nominal value. The accuracy of quantification was further verified using triplicate Quality Control (QC) samples prepared at 10 ppm, with an acceptance criterion of 85-115% recovery.

Table S9: Back-calculation for calibration curve of benzoic acid.

Calibration Curve of Benzoic acid (ppm)			
Standard level	Nominal Conc.	Back-Calculated Conc.	Accuracy (%)
STD 1	5	5.80	116.08%
STD 2	10	9.23	92.35%
STD 3	20	19.35	96.77%
STD 4	25	25.55	102.21%
STD 5	50	50.05	100.11%

Table S10: Back-calculation for calibration curve of propiophenone.

Calibration Curve of Propiophenone (ppm)			
Standard level	Nominal Conc.	Back-Calculated Conc.	Accuracy (%)
STD 1	2.5	2.61	104.43%
STD 2	5	4.87	97.39%
STD 3	10	9.86	98.57%
STD 4	20	20.06	100.31%
STD 5	25	25.18	100.71%
STD 6	50	49.92	99.84%

Also, triplicate QC samples prepared at 10 ppm were analysed. The results were ±10% of the nominal values.

Table S11: Verification using triplicate QC samples prepared at 10 ppm.

	Benzoic acid		Propiophenone	
	Calculated conc. (ppm)	Accuracy (%)	Calculated conc. (ppm)	Accuracy (%)
QC_1	9.82	98.17%	10.12	101.19%
QC_2	9.33	93.29%	10.13	101.29%
QC_2	10.16	101.63%	10.14	101.40%

The calibration curves for both analytes demonstrated excellent linearity. The back-calculated concentrations for all except one (5 ppm benzoic acid) standards were within ±10% of their nominal values, with most falling within ±5%, confirming the accuracy of the regression model. QC samples (10 ppm) yielded accuracies between 93.3% and 101.6%, well within the acceptance criteria.

Post-extraction addition

Additional post-extraction addition experiments were performed, by assessing the recovery via a post-extraction spike. Blank cotton and MOF samples were extracted, and the resulting supernatant was spiked with a standard solution to a final concentration of 20 ppm. Samples were analysed in triplicate to determine the percent recovery.

Table S12: Recovery from post-extraction spike for cotton.

Sample	Concentration (ppm)	
	Cotton with benzoic acid	Cotton with propiophenone
Post-spike_1	20.66	20.45
Post-spike_2	19.35	20.18
Post-spike_3	20.33	19.99

Table S13: Recovery from post-extraction spike for MOF.

Sample	Concentration (ppm)	
	MOF with benzoic acid	MOF with propiophenone
Post-spike_1	20.42	19.58
Post-spike_2	19.42	20.52
Post-spike_3	20.86	20.42

The post-extraction spike recovery was excellent for all material-analyte combinations, ranging from 97.1% to 104.3%, indicating that the extraction procedure was free from significant matrix effects.

Robustness tests

The robustness of the extraction procedure was evaluated by introducing deliberate minor variations to the method parameters: extraction solvent volume (RS) (9 mL and 11 mL vs. nominal 10 mL) and extraction time (RE) (27 min and 33 min vs. nominal 30 min). The results were compared against their respective nominal concentrations, with an acceptance criterion of ±10% deviation.

Table S14: Robustness tests for cotton samples.

Condition	Concentration (ppm)	
	Benzoic acid-infused cotton	Propiophenone-infused cotton
RS_11mL_1	18.30 (101.66%)	18.97 (105.39%)
RS_11mL_2	18.63 (103.50%)	18.87 (104.85%)
RS_11mL_3	17.28 (95.98%)	17.79 (98.81%)
RS_9mL_1	20.93 (95.12%)	21.07 (95.76%)
RS_9mL_2	21.82 (99.20%)	21.96 (99.82%)
RS_9mL_3	21.30 (96.84%)	23.16 (105.28%)
RE_27min_1	18.93 (94.64%)	19.38 (96.90%)
RE_27min_2	20.93 (104.63%)	20.45 (102.26%)
RE_27min_3	20.03 (100.15%)	20.70 (103.49%)
RE_33min_1	19.68 (98.42%)	19.28 (96.39%)
RE_33min_2	20.27 (101.33%)	20.12 (100.58%)
RE_33min_3	19.51 (97.55%)	19.67 (98.35%)

*RS_11mL: 11 mL of extraction solvent was used; nominal value was 18 ppm.
*RS_9mL: 9 mL of extraction solvent was used; nominal value was 22 ppm.
*RE_27min: The extraction time was 27 min; nominal value was 20 ppm.
*RE_33min: The extraction time was 33 min; nominal value was 20 ppm.

Table S15: Robustness tests for MOF samples.

Condition	Concentration (ppm)	
	Benzoic acid-infused MOF	Propiophenone-infused MOF
RS_11mL_1	18.43 (102.36%)	17.91 (99.52%)
RS_11mL_2	19.04 (105.78%)	18.85 (104.70%)
RS_11mL_3	18.34 (101.90%)	17.68 (98.21%)
RS_9mL_1	20.70 (94.09%)	21.00 (95.44%)
RS_9mL_2	21.84 (99.29%)	19.66 (98.28%)
RS_9mL_3	21.10 (95.90%)	19.38 (96.88%)
RE_27min_1	21.05 (105.24%)	20.98 (104.91%)
RE_27min_2	19.53 (97.60%)	19.66 (98.28%)
RE_27min_3	20.53 (102.67%)	19.38 (96.88%)
RE_33min_1	19.45 (97.27%)	19.92 (99.60%)
RE_33min_2	21.16 (105.82%)	19.64 (98.20%)
RE_33min_3	19.45 (97.27%)	19.78 (98.90%)

*RS_11mL: 11 mL of extraction solvent was used; nominal value was 18 ppm.
*RS_9mL: 9 mL of extraction solvent was used; nominal value was 22 ppm.
*RE_27min: The extraction time was 27 min; nominal value was 20 ppm.
*RE_33min: The extraction time was 33 min; nominal value was 20 ppm.

The method proved to be robust against minor variations in extraction time and solvent volume. All results under the modified conditions remained within ±10% of the expected nominal value, demonstrating the robustness of the method for routine analysis.

A carryover test was performed by injecting a blank solvent sample immediately following the injection of the highest concentration standard (50 ppm). No peaks were observed at the expected retention time ranges of benzoic acid or propiophenone. No carryover was detected, confirming adequate system cleaning between runs.

Table S16: Headspace analyte vapour content over pseudoscent-infused cotton by GC-MS.

Time	Peak area	
	Benzoic acid-infused cotton	Propiophenone-infused cotton
Day 0	887659	1099923
Day 1	1531749	8356049
Day 3	1627775	14047424
Day 7	1891451	24907392
Day 14	2108521	26245868

Table S17: Headspace analyte vapour content over pseudoscent-infused MOF by GC-MS.

Time	Peak area	
	Benzoic acid-infused MOF	Propiophenone-infused MOF
Day 0	697108	565723
Day 1	1070406	1325595
Day 3	1606988	10815208
Day 7	1667875	12672978
Day 14	1872646	19623738

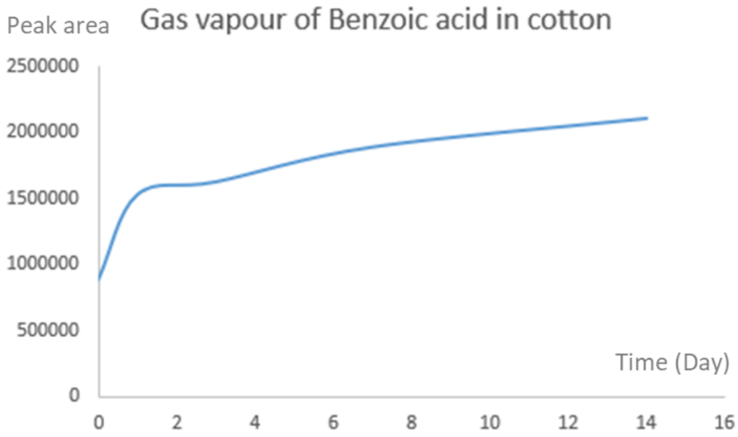

Fig. S13: Headspace concentration profile for benzoic acid-infused cotton.

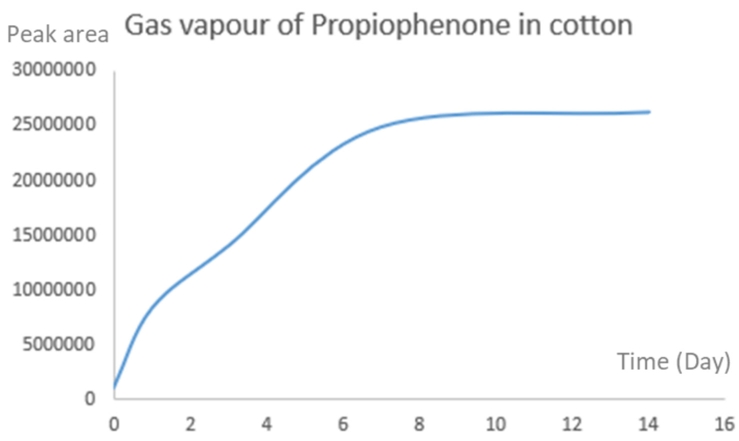

Fig. S14: Headspace concentration profile for propiophenone-infused cotton.

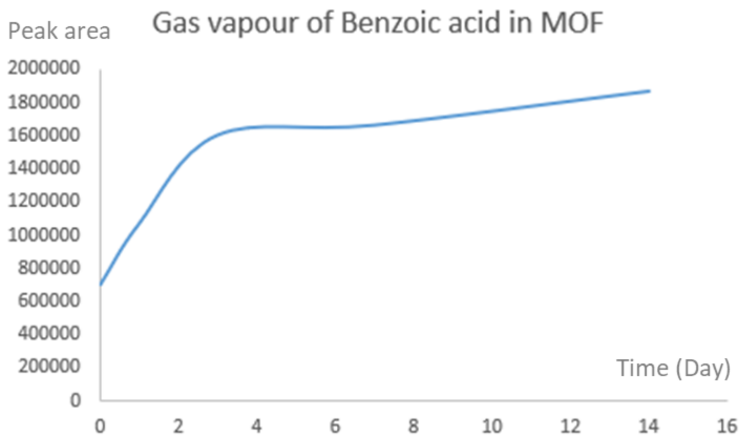

Fig. S15: Headspace concentration profile for benzoic acid-infused MOF.

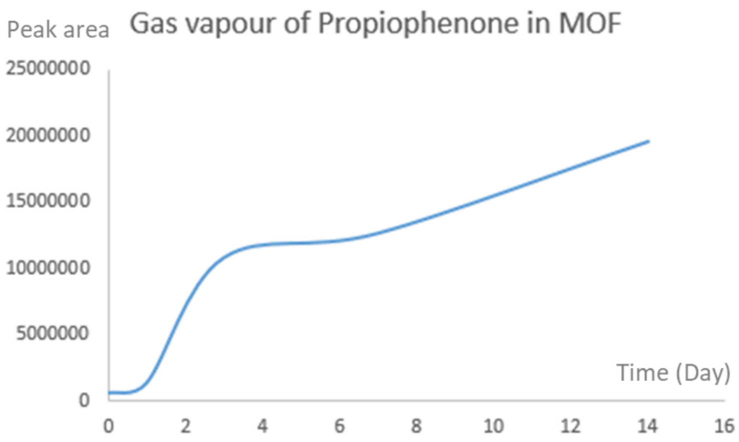

Fig. S16: Headspace concentration profile for propiophenone-infused MOF.

Table S18: Retained benzoic acid content in material by HPLC.

Time	Concentration (mg/g)	
	Benzoic acid-infused cotton	Benzoic acid-infused MOF
Day 0	17.05	13.61
Day 1	16.63	14.41
Day 3	18.43	13.45
Day 7	16.30	13.41
Day 14	17.40	13.13

The previously determined concentrations for benzoic acid-infused cotton and MOF after 14 days were (17.06 ± 0.13) mg/g and (13.67 ± 0.86) mg/g, respectively. The new results were within ±10% of the previous results.

Table S19: Retained propiophenone content in material by HPLC.

Day	Concentration (mg/g)	
	Propiophenone acid-infused cotton	Propiophenone-infused MOF
Day 0	4.06	1.57
Day 1	4.68	1.65
Day 3	4.26	1.56
Day 7	4.63	1.73
Day 14	4.25	1.69

The previously determined concentrations for propiophenone-infused cotton and MOF after 14 days were (4.11 ± 0.10) mg/g and (1.63 ± 0.03) mg/g, respectively. The new results were within ±10% of the previous results.

Table S20: Mass stability of cotton-based pseudoscent delivery systems.

Mass (g)	Storage time	Day 0	Day 1	Day 3	Day 7	Day 14
Benzoic acid	Before load (Day 0)	0.1009	0.1132	0.0987	0.1105	0.0999
	After load (Day 0)	0.1022	0.1153	0.0988	0.1120	0.1001
	After GC-MS (Day X)	0.1025	0.1150	0.0988	0.1122	0.1002
Propiophenone	Before load (Day 0)	0.1015	0.0988	0.0996	0.1000	0.1100
	After load (Day 0)	0.1012	0.0990	0.0999	0.1002	0.1103
	After GC-MS (Day X)	0.1010	0.0989	0.0996	0.1007	0.1102

No differences in mass of the cotton carriers were observed after 0-14 days of storage.
